# Two Points in the History of a Case

**Published:** 1880-06

**Authors:** 


### EDITORIAL.

---

### TWO POINTS IN THE HISTORY OF A CASE.

The failure of a physician to observe accurately the symptoms of a patient, so often leads to erroneous conclusions, as to be seldom deemed worthy of comment. An instance is referred to in the *Medical Record*, of May 15, which is so typical as to serve as a text for such a sermon as might be preached to many a well-meaning but misguided practitioner.

It appears that a Dr. Neftel, of New York, had conceived the notion of curing cataract by means of electricity; so he brought his batteries to bear upon the eyes of an old lady, whose crystalline lenses were partially opaque, but whose imaginative powers were of high order. The doctor, in reporting the case, says that "after each sitting the patient declared she could see more plainly. After one week, she declared herself able to read coarse print pretty well, and after two weeks she could read quite small print. After twenty-five applications the treatment was discontinued. The patient declared that not only had she recovered her earlier seeing power, but also needed weaker spectacles than before." It so happened that the lady was well known to the editor of a certain religious weekly, published in the same city, and this, together with another "wonderful cure of cataract," was forthwith paraded before its confiding subscribers. In one issue of this journal, the small corner vouchsafed to "Science," was entirely monopolized by an account of the almost miraculous results obtained by this skillful electrician. But unfortunately for ophthalmology and for the growing reputation of this gentleman, a couple of our most eminent oculists have published a statement, tending to throw discredit upon this brilliant triumph of galvanism.

In the number of the *Medical Record*, already alluded to, there is a letter signed by Doctors Agnew and Webster, in which the case in question is carefully reviewed, and in regard to which they say: "It will be observed that Mrs. M. had incipient cataract when she first came to see me on the 24th of July, 1878; that she had incipient cataract on the 14th of April, 1880. In the interval between those dates she had received galvanic treatment from Dr. Neftel. The vision of Mrs. M. was $\frac{20}{20}$ before the alleged cure, $\frac{20}{20}$ after the alleged cure, and sufficient to enable her to read Jaeger, No. 1, at fourteen inches on the 15th of April, 1880. The case is simply one of those common ones of stationary, or slowly increasing opacity of the lenses, and we must calmly wait for new light, if we are to change our belief that galvanism will not remove opacities of the crystalline lenses." A reply followed in the next number of the *Record*, in which Dr. Neftel charged the error in diagnosis upon the gentlemen mentioned. But as ill luck would have it, still another oculist entered the lists to cast greater suspicion upon the success of the method.

It was almost unkind thus to dampen the ardor of an enthusiastic electrician by such pointed references to dates, and to the details of exact measurement. But the moral is plain. If less reliance had been placed upon the statements of the patient and more upon careful investigation of the actual condition, the imaginary improvement would not have been credited. Moliere, by his drama of the *Malade imaginaire*, has treated many a Frenchman to a hearty laugh at the medical profession, and so must physicians expect to be ridiculed if they rely too implicitly upon the statements of their patients.

And while one phase of this case has in it a lesson to the physician, there is another which relates to our friends of the clerical profession. Just as the religious journal was quick to proclaim the wonderful cure, so are clergymen in general too ready to lend their credence and weight of authority to nostrums of every kind. There is hardly a patent medicine advertised, that cannot boast of testimonials from eminent divines. The degree of their "gullability" as to things medical, is simply astounding.

It is this hasty judgment from insufficient data—this morbid yearning for the miraculous, which makes skeptics in medicine as it has made skeptics in theology.

